# A national survey of pharmacists and interns in Aotearoa New Zealand: provision and views of extended services in community pharmacies

**DOI:** 10.1186/s12913-021-07158-w

**Published:** 2021-10-23

**Authors:** Janet McDonald, Caroline Morris, Megan Pledger, Phoebe Dunn, Ausaga Fa’asalele Tanuvasa, Kirsten Smiler, Jacqueline Cumming

**Affiliations:** 1 Health Services Research Centre, Victoria University of Wellington, PO Box 600, Wellington, 6140 New Zealand; 2 Department of Primary Health Care and General Practice, University of Otago, Wellington, PO Box 7343, Wellington, 6242 New Zealand

**Keywords:** Community pharmacy services, Pharmacists, Intern pharmacists, New Zealand, Survey, Health services research

## Abstract

**Background:**

Changes in pharmacy models of care, services and funding have been occurring internationally, moving away from the traditional dispensing role to more extended patient-facing roles utilising pharmacists’ clinical skills. This study aimed to identify the extended services offered by community pharmacy in Aotearoa New Zealand and the barriers and facilitators to extended services provision. The study is unique in that it includes intern (pre-registration) pharmacists.

**Methods:**

An online survey, conducted in 2018, of all pharmacists and intern (pre-registration) pharmacists working in a community pharmacy. Data were analysed using descriptive statistics and regression analyses.

**Results:**

The results are based on replies from 553 community pharmacists and 59 intern pharmacists (response rate: 19 and 26% respectively). Both pharmacists (83%) and interns (85%) want to work at the top of their scope of practice. Wide variation exists in the specific services individual pharmacists offer. Most pharmacists were accredited to supply the emergency contraceptive pill (95%), sildenafil for erectile dysfunction (86%) and trimethoprim for uncomplicated urinary tract infection (85%). Fewer were able to immunise (34%) or to supply selected oral contraceptives (44%). Just under a quarter could provide a Medicines Use Review (MUR) or Community Pharmacy Anticoagulation Management Service (CPAMS). Of the pharmacists not already accredited, 85% intended to gain accreditation to supply selected oral contraceptives, 40% to become vaccinators, 37% to offer CPAMS and 30% MUR. Interns expressed strong interest in becoming accredited for all extended services.

Poisson regression analyses showed key factors supporting the likelihood of providing extended services were owner and management support and appropriate space and equipment. Being excited about the opportunities in community pharmacy, having employer funding and time for training and sufficient support staff were also statistically significant.

**Conclusions:**

Pharmacists need time and a supportive management structure to enable them to deliver extended services. Health policy with a greater strategic emphasis on funding services and pharmacist training, and developing technician support roles, will help to minimise or eliminate some of the barriers to role expansion both in Aotearoa New Zealand and internationally.

**Supplementary Information:**

The online version contains supplementary material available at 10.1186/s12913-021-07158-w.

## Background

A strong primary health care system is recognised internationally as being essential for improving population health and health equity [1–3]. In Aotearoa New Zealand, health policy since 2001 has had a strong focus on improving access to primary health care services and reducing health inequalities (particularly for indigenous Māori, and for Pacific peoples), along with developing and fully utilising the health workforce [4–6]. New models of care have been developing, including in general practice services (e.g. [7]) and the Whānau Ora model which puts whānau (families) at the centre of integrated health, social and educational service delivery [8].

Alongside these broader primary health care system changes, changes in pharmacy models of care, services and funding have been occurring internationally, moving away from the traditional role of dispensing to more clinical or “cognitive” roles [9]. However, the availability of such services, and their remuneration, varies considerably between countries [10]. A renewed focus on quality use of medicines and extending the use of pharmacists’ skills in areas such as health promotion and chronic conditions management has the potential to improve both individual and population health outcomes [11, 12]. In addition, fully utilising the skills of the pharmacy workforce can contribute to addressing the growing burden of long-term conditions and reduce demands on other primary health care providers such as General Practitioners [11].

Developing pharmacists’ roles has translated into health policy both in Aotearoa New Zealand and internationally, and is supported by pharmacist professional stakeholder organisations across the globe [11, 13–17]. In practice, there has been a sea change in community pharmacy funding agreements (nationally agreed service contracts), with a shift away from payment for dispensing to greater remuneration for more clinical roles; Aotearoa New Zealand is similar in this regard to other jurisdictions such as Australia, Canada and the UK [18–21].

In Aotearoa New Zealand, about 75% of pharmacists work in about 1000 community pharmacies [11]. They are funded through the Integrated Community Pharmacy Services Agreement (ICPSA) [20], providing standard dispensing and professional advisory services, along with a growing range of clinical services. Table 1 illustrates some of the extended services most frequently offered by community pharmacists in Aotearoa New Zealand. The funded Long Term Conditions (LTC) Service was introduced by the government in 2012 to help people with multiple medications better self-manage and to improve medicines adherence [26]. Accredited pharmacists can deliver Medicines Use Reviews (MURs), Medicines Therapy Assessments (MTAs) and the Community Pharmacy Anticoagulant Monitoring Service (CPAMS) [22]. To gain accreditation, pharmacists must complete an established, mandatory training course for each specific service requiring this. Training costs and training provider organisations vary and may be funded by individual pharmacists or paid by their employer.

**Table 1:** Extended services offered by community pharmacists in Aotearoa New Zealand [22–25]

Service	All registered pharmacists	Accredited pharmacists
Long term conditions service – LTC (optimising supply and use of prescribed medicines)	√	
Medicines use review – MUR (optimising medicines understanding and adherence)		√
Medicines therapy assessment – MTA (optimising effective medicines use)		√
Community pharmacy anticoagulation management service – CPAMS (INR testing and warfarin adjustment)		√
Prescription medicines except in defined circumstances when supplied by a pharmacist who has completed mandatory training		
• Emergency contraceptive pill		√
• Sildenafil		√
• Selected oral contraceptives		√
• Trimethoprim		√
Immunisation (currently influenza, Tdap, zoster, meningococcal, MMR, COVID-19)		√

In tandem with the above changes, in more recent years, Aotearoa New Zealand has led the way in the re-classification of some medicines that were previously only available on prescription [27]. This has enabled the controlled supply of a medicine by an accredited pharmacist under a “user-pays” model. Examples include the supply of sildenafil for erectile dysfunction [23] and trimethoprim for uncomplicated urinary tract infections [28]. Legislative changes have also enabled pharmacists to administer some vaccinations, including influenza vaccines following vaccinator training [24]. In 2020, registered intern pharmacists (graduates who have completed their pharmacy degree and are undertaking a year’s mandatory pre-registration training prior to obtaining their practising certificate) were approved to be able to administer influenza vaccinations after successfully completing vaccinator training [29].

Some extended services are available nationally (LTC, along with immunisation and controlled supply of medicines, provided a pharmacy has accredited pharmacists). However, other extended pharmacy services are locally commissioned and funded in a defined geographical area by the local District Health Board. Medicines use review, medicines therapy assessment and the community pharmacy anticoagulation management service require a local contract and are therefore not universally available. Another potential constraint on the availability of extended services is having sufficient private space within the pharmacy. Some jurisdictions require a private consultation room for extended services (e.g. the NHS England New Medicine Service for flu vaccinations [30, 31]). The ICPSA requires respect for service users’ privacy, and for CPAMS specifically, access to a private area for testing and counselling [20], with private consultation rooms becoming more common in community pharmacies generally. For additional context, a summary of the national ICPSA schedules detailing the services that each schedule encompasses is shown as an Additional file 1.

Aotearoa New Zealand data on the extension of cognitive roles in community pharmacy is scarce [32, 33]. A survey of all community pharmacies in Aotearoa New Zealand was undertaken in 2014/15 [33], approximately 2 years after the introduction of a significant remuneration change in the national Community Pharmacy Services Agreement, to identify whether this had translated into changes in the delivery of more patient-centred services. While a wide range of different activities were identified, unsurprisingly, the most commonly offered were the mainstream services of education and advice about medicines and prescription dispensing. The main barriers to introducing new services were financial cost and lack of staff. Another survey of all primary care pharmacists, published in 2017, identified limited provision of cognitive services by community pharmacists. This survey did not, however, capture comprehensive information on specific services offered [32].

Lack of time, staff and remuneration are regularly cited in the literature by pharmacists as barriers to extended care [34–38], while a private consultation space is often identified as a facilitator of extended care [34, 36]. Variation in the availability and quality of service provision has also been noted [35, 37, 38].

The 2014/15 New Zealand survey by Smith et al. [33] was undertaken at the pharmacy, rather than the pharmacist, level and was therefore unable to fully uncover issues specific to individual pharmacists. Furthermore, pharmacy services have been continuing to develop and expand in Aotearoa New Zealand since 2014 and we know little about the extent to which this is occurring successfully and what the enablers and barriers to further progress might be. A notable absence in previous research, both in Aotearoa New Zealand and internationally, is the views of intern pharmacists, whose intentions for future practice are important to understand.

The survey reported in this paper forms part of a larger project which aims to understand current developments in community pharmacy services in Aotearoa New Zealand; how community pharmacy strategies are being implemented and the response of pharmacists, service users and other health practitioners; and what contexts may support successful implementation. As extended services by community pharmacists are gaining traction in many other jurisdictions [9], our findings are likely to be of high relevance for health policy internationally.

## Methods

The focus of the survey was the new and expanded services being developed and offered in community pharmacy in Aotearoa New Zealand. Questions were developed from the literature and research team knowledge of recent service developments, with advice from our advisory groups (including representatives from policy, pharmacy, medicine, nursing, consumer and indigenous sectors). The survey was created online using Qualtrics software [39] and piloted by 4 practising pharmacists; minor changes were then made to improve clarity.

The survey was available from 13 February to 19 March 2018 and open to all pharmacists and intern pharmacists working in community pharmacies. An initial invitation to participate was sent out through the weekly e-newsletter of the Pharmaceutical Society of New Zealand (PSNZ), the professional body for Aotearoa New Zealand pharmacists. At the time the survey was undertaken, the e-platform through which pharmacists were required to record and submit their mandatory professional development records for continued registration was only available through PSNZ. The invitation to participate provided information about the survey and an electronic link. Three reminders were sent by PSNZ before the survey closed.

After reading information about the survey and consenting to participate, respondents were asked if they worked in a community pharmacy as a pharmacist (routed to the main section of the survey) or intern pharmacist (routed to the relevant subsections of the survey).

Most questions offered choices of response (single and multiple choices possible) and occasionally a numeric answer. Some questions used Likert scales. Optional text boxes allowed respondents to add further comments or answers that did not fit with the range of choices provided.

We decided that no respondent should be forced to answer any question with the hope that it would aid respondent retention. The implied assumption is that no respondent should be removed from the analysis group if they did not provide complete information for the survey. This allowed respondents to skip some questions and so generate missing values for any question they skipped. In this case, a respondent reading a question and choosing not to answer that question provided some information about the respondent and these respondents were grouped under the option “Did not answer”.

There were some respondents who left the survey before completing it and they were also included in the analysis group. However, these respondents were not included in the analysis of questions that occurred after they left the survey. As they did not read any further questions, we have no information about how they would have answered those questions. Dropping them from the analyses for those questions is identical to assuming that the respondents who left before completion of the survey would give similar answers to those that did answer those particular questions. Approximately 8% of pharmacists and 5% of interns who started the survey did not complete it. The tables are annotated with the percentage of community pharmacists who had left the survey when the particular questions were asked. For conciseness, this is not included for interns, who were slightly more likely to persist.

This method here is different to standard practice in internet surveys which is to make every question compulsory to answer and to exclude all respondents who did not finish the survey. However, we feel that this practice fails to capture all the information from those who chose to respond.

The survey was responded to by pharmacists, but the research questions related to both pharmacists and pharmacies. As pharmacists from highly staffed pharmacies would provide more information about their pharmacy than pharmacists from sole-charge pharmacies, weights were developed based on the inverse number of pharmacists that a pharmacist said worked in his or her pharmacy. The responses from the pharmacists using these weights were used to generate statistics pertaining to the pharmacy.

A count was made of extended services reported by each pharmacist. This was used as the response in Poisson regression models where the independent variables were perceptions about themselves and the environment of their pharmacy. The independent variables required answers on a five-category Likert scale from strongly agree to strongly disagree plus the option “don’t know”. For the purposes of this question, responses on the continuum were analysed with those who strongly agreed or agreed grouped together and a second group made up of the remainder. The question that asked respondents about their level of agreement with “I have the business skills and financial knowledge to manage and develop my business in the current environment” was analysed and presented for those respondents who said they were pharmacist owners or pharmacist managers.

Relative rate estimates (RR) were calculated and *p*-values were presented that test whether the relative rate was different to 1. A RR greater than 1 meant that respondents who agreed or strongly agreed with the statement were offering more services, a RR less than 1 means those who agreed or strongly agreed were offering fewer services compared with those who did not agree with the statement.

## Results

### Response rate

There were 553 pharmacists and 59 intern pharmacists working in a community pharmacy who consented and undertook their stream of the survey with 508 pharmacists and 56 intern pharmacists completing the survey. In 2018, there were 2958 community pharmacists [40] and 225 interns (D. Wallace, PSNZ, personal communication), giving response rates of 19 and 26% respectively.

### Demographic information

Table 2 shows 67% of respondent pharmacists were female (approximately the same as national workforce data [40]), with representation across the age span. As expected, intern respondents were younger and 75% were female (compared with 67% in their year group; D. Wallace, PSNZ, personal communication). While most pharmacists were New Zealand European (68%), a high proportion of interns were Asian (64%), reflecting the changing demography of Aotearoa New Zealand pharmacists, with the fastest growing ethnic group being Asian pharmacists [40]. Four percent of both pharmacist and intern respondents were Māori (2% in workforce demographic data, but far below their population proportion of about 17%; the low proportion of Pacific pharmacists likewise underrepresents the national population [40–42]).

**Table 2:** Demographics

	Pharmacists		Interns	
	***n*** = 553		***n*** = 59	
*Gender (column %)*				
Female	67	(66^a^)	75	(67^b^)
Male	33	(34 ^a^)	23	(33^b^)
Did not answer	< 1		2	
*Age* (column %)^c^				
< 25 years	8		86	
25–34 years	31		13	
35–44 years	17		0	
45–54 years	19		0	
55–64 years	17		0	
65+ years	5		0	
Did not answer	3		2	
*Ethnicity* (%; multiple responses allowed)				
New Zealand European	68	(56^d^)	39	(30^e^)
Māori	4	(1^d^)	4	(4^e^)
Pacific	< 1	(1^d^)	0	(1^e^)
Asian	27	(26^d^)	64	(41^e^)
Other	4	(16^d^)	2	(18^e^)
Did not answer	< 1		0	(6^e^)
*Years working as a pharmacist* (column %)				
0 - < 5	21			
5 - < 10	17			
10 - < 20	20		NA	
20 - < 30	16			
30 - < 40	19			
40+	8			
*Likely to leave community or primary health care pharmacy within the next 5 years (column %)*				
Yes	37		34	
No	62		66	
Did not answer	1		0	

Notes: a) 2018 workforce demographic data [40] b) 2018 intern year group (D. Wallace, PSNZ, private communication) c) no comparable workforce data available; d) 2018 workforce demographic data (primary ethnicity only) [40]; e) 2018 intern year group (primary ethnicity only) (D. Wallace, PSNZ, private communication; f) Of the 553 pharmacists answering the survey, 8.1% had left the survey before the section on demographics

Thirty-seven percent of pharmacists said they were “likely to leave community or primary health care pharmacy within the next 5 years”. Of those who provided a reason, about 30% were intending to retire. Other reasons included work stress, lack of job satisfaction and concern about the future of pharmacy. One third of interns (34%) were considering leaving the sector within the next 5 years; half of those giving a reason were planning to shift to another area of pharmacy work or further their education, while about a quarter were unhappy about the direction of pharmacy or considering another career path.

Weighted results showed that pharmacies had an average of 2.6 pharmacists and 1.7 technicians, with few having an intern or pharmacy accuracy checking technician (Table 3). Just over half the pharmacies were in a major city with almost half within or next door to a health centre and about a third in a central city/town shopping area or suburban shopping centre. The vast majority (84%) were independently owned and 41% belonged to a branded retail group. Operating as a franchise of a national brand has the potential to enable access to more competitive purchasing power and additional professional support [43]. Given current levels of funding for community pharmacy, 9% of all pharmacists thought the pharmacy was thriving, 63% thought it was getting by, while 19% considered it unable to survive long-term. Owners and managers were slightly more likely to say the pharmacy was getting by (70%).

**Table 3:** The community pharmacy

	Pharmacy^**a**^	
***Number of staff (including respondent)*** (mean and range)^b^		
Pharmacists	2.6	(1–22)
Interns	0.3	(0–14)
Pharmacy Accuracy Checking Technicians	0.1	(0–6)
Other technicians	1.7	(0–15)
***Pharmacy location A*** (column %)^c^		
Main centre (5 major cities)	53	
Urban area, pop’n 30,000+	20	
Urban area, pop’n 10,000-29,000	11	
Urban area, pop’n 1000-9999	12	
Rural area, pop’*n* < 1000	3	
Did not answer	< 1	
***Pharmacy location B*** (%; multiple responses allowed)^b^		
Within or next door to a health centre	46	
In a central city or town shopping area	32	
In a suburban shopping area	34	
Supermarket	4	
Part of Māori health service or in close proximity to a Māori health service/ provider and/or marae community	8	
Part of Pacific health service or in close proximity to a Pacific health service/ provider and/or Pacific community	2	
Other	7	
Did not answer	< 1	
***Pharmacy ownership*** (column %)^b^		
Independent	84	
Corporate	14	
Other	3	
Did not answer	< 1	
***Part of a branded pharmacy retail group*** (column %)^b^		
Yes	41	
No	59	
Did not answer	< 1	
***Financial sustainability – Given current levels of funding for community pharmacy, would you describe this community pharmacy as:*** (column %)^d^		
Thriving	9	
Getting by	63	
Unable to survive long-term	19	
Don’t know	10	
Did not answer	< 1	

Note: a) Based on weighted responses of pharmacists; b) Of 554 community pharmacists, < 0.2% had left the survey at this point; c) Similarly, 8.1% and d) 7.0% of community pharmacists had left the survey at this point. They are not included in the analysis

### Extended services

There was considerable variation in extended services able to be offered by pharmacists. Almost all pharmacies (96%) offered the LTC service: 21% had 1–49 people enrolled; 18% had 50–99 enrolments; 41% had 100 or more enrolments; while 17% didn’t know. Table 4 shows the majority of pharmacists were accredited to offer the emergency contraceptive pill (ECP) (95%), sildenafil for erectile dysfunction (86%) and trimethoprim for uncomplicated urinary tract infection (85%). Fewer were able to give immunisations (34%) or to supply selected oral contraceptives (44%). Just under a quarter could provide a MUR or CPAMS while only 3% could undertake a MTA. Of the pharmacists not already accredited, 85% intended to be able to supply selected oral contraceptives in future and there was further interest in future accreditation for immunisation (40%), MUR (30%) and CPAMS (37%). Interns expressed strong interest in becoming accredited for all extended services, with MTA the least likely at 65%.

**Table 4:** Extended services

Service	Pharmacists currently accredited (%)	Pharmacists not currently accredited, but intending to gain accreditation in future (%)^a^	Interns intending to gain accreditation in future (%)^a^
Emergency contraceptive pill	95	62 (3)	95 (0)
Sildenafil	86	57 (14)	91 (2)
Trimethoprim	85	78 (8)	78 (8)
Supply selected oral contraceptives	44	85 (7)	91 (5)
Immunisation	34	40 (22)	82 (12)
Medicines use review	23	30 (26)	77 (16)
Community pharmacy anticoagulation monitoring service	23	37 (26)	75 (19)
Medicines therapy assessment	3	20 (34)	65 (28)
Other	15	3 (16)	12 (19)

Notes: a) Percentage reporting ‘Yes’ with percentage reporting “Don’t know” in brackets; b) Of 554 community pharmacists, 1.4% had left the survey at this point and are not included in the analysis

Other extended services commonly provided in the pharmacy (Table 5) included supply of nicotine replacement therapy (90%) and smoking cessation counselling (74%), blood pressure measurement (81%), opioid substitution therapy (68%) and the clozapine service monitoring (54%). Among those not already providing the service, 37% were interested in supplying nicotine replacement therapy and 30% smoking cessation counselling in future, but less than 20% expressed an intention to provide any of the other services (with 33–55% saying they did not know).

**Table 5:** Other services currently provided by the pharmacy

Service	Currently provided by the pharmacy (%)^a^	Intend to provide in future (%)^a,b^
Supply of nicotine replacement therapy	90 (2)	37 (33)
Blood pressure	81 (< 1)	16 (47)
Smoking cessation counselling	74 (4)	30 (43)
Opioid substitution therapy	68 (< 1)	9 (37)
Pharmacy clozapine service	54 (< 1)	14 (36)
Blood glucose	23 (3)	14 (52)
Vitamin B12	10 (3)	11 (53)
Group A streptococcus	9 (3)	12 (52)
Cholesterol	7 (3)	11 (50)
Bone density	6 (3)	6 (47)
HbA1c	2 (3)	10 (55)
Iron	1 (3)	10 (53)
Other	4 (9)	< 1 (23)

Notes: a) Percentage reporting ‘Yes’ with percentage reporting “Don’t Know” in brackets; b) Only asked of those not already providing the service; i.e. additional provision; c) Of the 554 community pharmacists, 1.4% had left the survey at this point

### Enablers and barriers to extended service provision

Table 6 shows the prevalence of agreement for each factor that could influence the provision of extended services by pharmacists. There was strong agreement among both pharmacists (83%) and interns (85%) that they wanted to work at the top of their scope of practice and most pharmacists believed the pharmacy owner and pharmacy management supported providing more services (70 and 68% respectively). However, while 72% of interns were excited by the opportunities in community pharmacy, only 56% of pharmacists agreed.

**Table 6:** Enablers and barriers to offering extended services

Percentage of respondents who Agreed or Strongly Agreed to each Statement (%)		
	Pharmacists	Interns
***Attitudes and support for extended services***		
I want to work at the top of my scope of practice	83	85
The pharmacy owner supports providing more services	70 (6)	–
The pharmacy management supports providing more services	68 (7)	–
I am excited by the opportunities in community pharmacy	56	72
I’m too busy dispensing to offer more services	46	–
I’m happy with my current work roles and don’t want to provide more services	20	–
***Skills and training***		
I need additional training in order to offer additional services	86	86 (4)
I feel culturally competent to deliver pharmacy services	80	73
My employer provides funding for me to undertake training and accreditation	57	–
My employer allows me time to undertake training and accreditation	53	–
I can afford training and accreditation	42	–
I have the business skills and financial knowledge to manage and develop my business in the current environment	37 (4)	–
***Resources and infrastructure***		
The pharmacy has a suitable private consultation area to talk with people confidentially	82	–
The pharmacy has enough equipment or other resources to enable it to provide more services (e.g. fridge space to store vaccinations)	65	–
I have access to relevant patient health information from other health providers	54	–
The pharmacy can access funding for more services	25 (23)	–
There are sufficient technicians or other support staff to free up my time to offer more services	24	–
The available funding covers the cost of providing the service	11 (19)	–
***Consumers and local relationships***		
There is consumer demand for more services	64 (4)	–
I’m concerned about the impact on my relationships with other health providers if I provide more services	38	25 (5)
Consumers can afford to pay for additional services	30 (4)	–
Consumers are willing to pay for additional services	26 (4)	–

Notes: 1) Interns answered a subset of these questions. Dashes indicate they were not asked this question. 2) Figures in brackets are the percentage of people who said “Don’t Know” or did not answer but had not left the survey and they are reported if they sum to 4% or more; c) Of the 554 community pharmacists, 5.4% had left the survey at this point

Most pharmacists (80%) and interns (73%) felt culturally competent to deliver services. Among both pharmacists and interns, 86% said they needed additional training in order to offer additional services. Among pharmacists, 57% said their employer provided funding and 53% that their employer allows time for them to undertake training and accreditation, but only 42% said they could afford training and accreditation.

The pharmacy usually had a private consultation space (82%) and about two-thirds of pharmacists considered there was enough equipment or necessary resources to provide more services, with just over half agreeing they had access to relevant patient health information from other health providers. Few pharmacists (11%) agreed that the available funding covers the cost of providing services or the pharmacy can access funding for more services (25%). Only 24% of pharmacists thought there were sufficient technicians or other support staff to free up their time for other services.

There was strong agreement among pharmacists that there is consumer demand for more services (64%) but much less agreement that consumers can afford (30%) or are willing (26%) to pay for additional services. Some pharmacists (38%) and interns (25%) agreed they were concerned about the impact that providing more services would have on their relationship with other health providers.

Table 7 shows relative rate estimates and 95% confidence intervals of the association between attributes of the pharmacy or pharmacist and the likelihood of offering extended services. Of the significant results, pharmacists offered more services if they strongly agreed or agreed that the pharmacy owner (RR 1.19, *p* < 0.001) or management (RR 1.18, *p* < 0.01) supported providing more services, or that the pharmacy had enough equipment or other resources to enable it to provide more services (RR 1.19, *p* < 0.001). Pharmacists offered more services, on average, if they agreed or strongly agreed that they had a suitable private consultation area (RR 1.13, *p* < 0.05); sufficient technicians or other support staff (RR 1.12, *p* < 0.05); employer funding (RR 1.12, *p* < 0.05) and time (RR 1.09, *p* < 0.05) for training and accreditation; and consumer demand for more services (RR 1.10, *p* < 0.05). Pharmacists who were ‘excited by the opportunities in community pharmacy’ (RR 1.12, *p* < 0.05) also provided more services.

**Table 7:** Unadjusted Poisson regression analyses of the association between attributes of the pharmacy or pharmacist and the likelihood of offering extended services

	Relative Rate	95% Confidence Interval^a^
***Attitudes and support for extended services***		
I want to work at the top of my scope of practice	1.09	(0.97,1.22)
The pharmacy owner supports providing more services	1.19	(1.08,1.32)
The pharmacy management supports providing more services	1.18	(1.06,1.30)
I am excited by the opportunities in community pharmacy	1.12	(1.02,1.22)
I’m too busy dispensing to offer more services	0.93	(0.86,1.01)
I’m happy with my current work roles and don’t want to provide more services	0.94	(0.84,1.04)
***Skills and training***		
I need additional training in order to offer additional services	0.90	(0.80,1.01)
My employer provides funding for me to undertake training and accreditation	1.12	(1.03,1.22)
I feel culturally competent to deliver pharmacy services	1.08	(0.97,1.21)
My employer allows me time to undertake training and accreditation	1.09	(1.01,1.19)
I can afford training and accreditation	1.07	(0.99,1.17)
I have the business skills and financial knowledge to manage and develop my business in the current environment	0.95	(0.84,1.07)
***Resources and infrastructure***		
The pharmacy has a suitable private consultation area to talk with people confidentially	1.13	(1.01,1.27)
The pharmacy has enough equipment or other resources to enable it to provide more services (e.g. fridge space to store vaccinations)	1.19	(1.09,1.31)
I have access to relevant patient health information from other health providers	1.06	(0.97,1.15)
The pharmacy can access funding for more services	1.01	(0.91,1.11)
There are sufficient technicians or other support staff to free up my time to offer more services	1.12	(1.02,1.24)
The available funding covers the cost of providing the service	1.02	(0.90,1.17)
***Consumers and local relationships***		
There is consumer demand for more services	1.10	(1.00,1.20)
I’m concerned about the impact on my relationships with other health providers if I provide more services	0.99	(0.91,1.08)
Consumers can afford to pay for additional services	1.07	(0.98,1.17)
Consumers are willing to pay for additional services	1.10	(1.00,1.21)

Notes: a) Wald confidence intervals on the log scale which have been exponentiated

## Discussion

This survey has shown a wide variation in the specific extended services offered by individual pharmacists in Aotearoa New Zealand. The key factors identified as supporting the likelihood of providing extended services are, owner and management support and appropriate space and equipment. Being excited about the opportunities in community pharmacy, having employer funding and time for training, and sufficient support staff were also significant.

The level of extended services offered by pharmacist respondents was variable. Most can offer services that require accreditation and target a specific medicine or medicine class for a specified therapeutic reason e.g. ECP, sildenafil and trimethoprim. The uptake of the supply of selected oral contraceptives was lower; however, this is unsurprising given that the mandatory training course had only been for available for approximately 6 months when the survey was issued [44]).

The uptake of the LTC service, which is part of the national contract and does not require completion of an accreditation training course to deliver, was high. However, the uptake of services requiring accreditation that target patients’ medicines and medicine-taking behaviour in greater depth and breadth, for example, MUR and MTA, were far lower. It is notable that in contrast to other services, MTA accreditation requires more intensive training/work via submission of a portfolio of evidence, and this could explain the low uptake.

Uptake of immunisation and CPAMS were also relatively low. Training courses vary in terms of provider, financial cost and time commitment to undertake. Immunisation requires mandatory training by the national Immunisation Advisory Centre [24]. A New Zealand survey of pharmacists’ views about administering vaccines found strong support for doing so, but among those who had not yet undertaken vaccination training, cost (both for set up and for training) was the most common reason given [45]. To offer the CPAMS service, pharmacists need formal training and to purchase the required equipment. In addition, the availability of local service delivery contracts and a good relationship with the general practice of patients enrolled in the CPAMS service are vital [46, 47]. With 38% of the pharmacists in our survey and a quarter of the interns ‘concerned about the impact on my relationships with other health providers if I provide more services’, this could be a barrier to fully utilising pharmacists’ skills. Relationships and/or attitudes of doctors have been previously identified internationally as having an impact on the provision of extended services [34, 36, 37].

These results align with previous Aotearoa New Zealand research of services offered by *pharmacies* in 2014 [33], in which services were ranked from 1 (provided by most pharmacies) to 56. The LTC service ranked 4, ECP 8, trimethoprim 10, MUR 25, immunisation 30, CPAMS 35, and MTA 46.

While there was varying intention among pharmacist respondents for offering a service in the future, intern pharmacists were very keen to be offering extended services when they held a practising certificate or were legally able to do so. This may reflect to some degree a lack of awareness of the financial cost and other requirements for accreditation for services where the user pays (e.g. trimethoprim), or a lack of insight into the vagaries of the local commissioning of contracts for other services e.g. MUR, CPAMS. However, it could also indicate changing expectations about the role of pharmacists: Butterworth et al. [48] noted greater enthusiasm for training and extended roles among recently qualified pharmacists than by those with more experience in primary care. It is also likely that the current suite of extended services will develop and change in future (for example, the need for CPAMS may reduce as newer anticoagulants replace warfarin).

It was notable that of the services that are currently routinely offered in Aotearoa New Zealand, most are for physical health conditions. This aligns with the existing literature where services are offered more frequently for physical conditions than mental health care [34]. The mental health services currently funded at a national level in Aotearoa New Zealand community pharmacies are the clozapine monitoring service (aimed at patients with severe mental illness) and the opioid substitution therapy service (for the treatment of addiction). Indeed, of all the “other” services self-identified by respondents as being offered, a very limited number encompassed mental health. These were focused exclusively on selective serotonin re-uptake inhibitor counselling (SSRI – an antidepressant medication), a service that is funded in only some regions of the country. Primary mental health care is an area where community pharmacy could have an increased and valuable role to play internationally [11, 13, 49], especially given the incidence of mild to moderate mental illness is rising, and this is being further exacerbated as a result of COVID-19 [50–52]. Furthermore, the profile of community pharmacists has already been highlighted due to their role as essential workers during COVID-19 lockdowns internationally. The pandemic is also promoting greater use of community pharmacy as a setting for immunisation. For example, NHS England has made COVID-19 vaccination a local enhanced service for community pharmacy, with 600 pharmacies offering the service in May 2021 [53, 54] and a growing number of New Zealand community pharmacies have become involved in the COVID-19 general population vaccination programme, since July 2021 [55].

Many of the barriers to service delivery identified in the present study have been widely reported over many years in the international literature. Lack of time, staff and funding are issues that are regularly cited [33–38] and were also found in this study. With respect to time and staffing, pharmacists offered more services if they had sufficient technicians or other support staff. A recent innovation in Aotearoa New Zealand has been the introduction of the pharmacy accuracy checking technician role [56]. This role, which has also developed in the US and the UK [57, 58], aims to release pharmacists from a predominantly dispensing role to spend more time on other clinical activities and services. However, it was notable that very few pharmacists in the present study worked with a technician trained in this advanced role.

With respect to funding, only a small minority of respondents thought the available funding covered the costs of providing services and while the majority agreed there is consumer demand for more services, most did not think consumers were willing or able to pay for additional services. Others have previously reported the attitudes and perceptions of the public as important [35, 36], with a lack of interest, lack of uptake or willingness to pay identified [33, 34, 37, 59].

Our study highlights that one of the most important factors supporting the likelihood of providing extended services in Aotearoa New Zealand is owner and management support. The impact that an owner can have on the time available to support extended services by pharmacist employees has been recently reported in the context of the delivery of primary mental health care [60]; this is very likely to also hold true for the delivery of any extended service. The “culture” within a pharmacy undoubtedly has an important role to play [36, 38].

Most pharmacists and interns wanted to work at the top of their scope of practice, but fewer were excited by the opportunities in community pharmacy. It is not unexpected that some pharmacists approaching the end of their career were considering leaving the profession in the short to medium-term due to retirement. However, it is concerning for the profession that some intern pharmacists were unhappy about the direction of pharmacy or considering another career path. This is something that needs to be urgently addressed at policy and professional stakeholder level. Given that pharmacy undergraduate students and intern pharmacists are the future of the profession, it is important that, internationally, their expectations of the role are met. Future research should investigate this issue further. We also note the underrepresentation of Māori and Pacific pharmacists in the profession, as with other health professions in Aotearoa New Zealand; addressing this is a priority for the health workforce [61].

Community pharmacists are a highly skilled workforce. Both professional pharmacy organisations and health policy documents internationally have highlighted the value of an extended role for pharmacists within the wider primary health care arena [14–17, 62, 63]. However, the potential of this workforce will only be able to be fully realised by working as part of collaborative and integrated primary health care teams [6, 11, 64–66].

The enablers and barriers identified in this survey are being explored further in a series of case studies where the services offered can be matched to the context in which they are delivered. This will enable us to ascertain how the traits of individual pharmacy staff, organisational constraints and the local community environment intersect and impact on service expansion and delivery.

### Strengths and limitations

A strength of this survey is that it included both practising community pharmacists and intern pharmacists. As the future of the pharmacy profession, the views of intern pharmacists are vitally important to capture but have very rarely been explored. While the response rate is low and therefore limits the generalisability of the results, it is comparable to other primary care pharmacy surveys [32, 67] and we have shown how our sample compares with the demographics of the profession identified through other sources. We have included respondents in this analysis even if they did not fully complete the survey and by the end of the survey this accounts for 8.1% of the 553 community pharmacists originally in the sample. However, standard practice is to discard the responses of all respondents who did not fully complete the survey and so, by keeping respondents, we feel that our analysis is more thorough. This study is part of a longitudinal survey which will be repeated in 2022 to ascertain whether there has been a shift in roles and whether uptake of services has been increasing over time.

There was some degree of missing data in the survey as respondents did not have to answer any question and could exit the survey at any time (although they could return to continue to complete it if they wished). This meant that some questions had fewer responses and that the missingness was more obvious at the end of the survey than at the beginning. While this missingness cannot be overcome, we have reported how we handled this issue to give the best estimates possible.

## Conclusions

Many community pharmacists and intern pharmacists are interested in offering more clinical services but need time and a supportive management structure to release them from the core task of dispensing and to facilitate the completion of the specific training prerequisites. Health policy with a greater strategic emphasis on funding extended services and training, and developing technician roles to free up pharmacists’ time, should act as a lever to direct focus and help to minimise or eliminate some of the barriers to role expansion that currently exist both in Aotearoa New Zealand and internationally. The pharmacy profession also needs to continue to take opportunities to develop their services and champion the contribution they can make to primary health care services.

## Supplementary Information

**Additional file 1: Appendix 1.** Integrated Community Pharmacy Services Agreement (2020) Service Schedules.

## Data Availability

The datasets generated and/or analysed during the current study are not publicly available as ethics approval was given on condition that only members of the research team would have access to the data and the data would not be shared.
