# Supplementary material for: A national survey of pharmacists and interns in Aotearoa New Zealand: provision and views of extended services in community pharmacies
Source: BMC Health Serv Res. 2021 Oct 23;21:1147. doi: 10.1186/s12913-021-07158-w (PMC8540874; doi:10.1186/s12913-021-07158-w)
Supplement: Supplementary file 1 — Additional file 1: Appendix 1. Integrated Community Pharmacy Services Agreement (2020) Service Schedules. [file 12913_2021_7158_MOESM1_ESM.docx]

A national survey of pharmacists and interns in Aotearoa New Zealand:

provision and views of extended services in community pharmacies

**Appendix 1**

**Integrated Community Pharmacy Services Agreement (2020) Service Schedules**

| Nationally-consistent services offered to all providers:   1. Schedule 1 (Dispensing services and professional advisory services) 2. Schedule 2 (Additional professional advisory services) |
| --- |
| Schedule 3A:  Nationally-consistent services schedules that may be offered to all or some providers   - 3A.1 Opioid substitution treatment services - 3A.2 Aseptic services - 3A.3 Sterile manufacturing services - 3A.4 Clozapine services - 3A.5 Influenza immunisation services - 3A.6 Measles, mumps and rubella immunisation services |
| Schedule 3B:  Locally commissioned services   - 3B.1 Long term conditions pharmacy services - 3B.2 Community residential care pharmacy services - 3B.3 Age-related residential care pharmacy services - 3B.4 Special foods services - 3B.5 Community pharmacy anti-coagulation management services (CPAMS) - 3B.6 Smoking cessation services |
| Schedule 3C:  Any additional services agreed between a DHB and a provider |

Source:

TAS. Integrated Community Pharmacy Services Agreement 1 October 2020. <https://tas.health.nz/dhb-programmes-and-contracts/community-pharmacy-programme/icpsa/>. Accessed 27 May 2021.
